# Fruitful Brewing: Exploring Consumers’ and Producers’ Attitudes towards Beer Produced with Local Fruit and Agroindustrial By-Products

**DOI:** 10.3390/foods13172674

**Published:** 2024-08-24

**Authors:** Nazarena Cela, Michele Filippo Fontefrancesco, Luisa Torri

**Affiliations:** 1 University of Gastronomic Sciences, 12042 Bra, Italy; n.cela@unisg.it (N.C.); or michele.fontefrancesco@unicatt.it (M.F.F.); 2 Department of Sociology, Università Cattolica del Sacro Cuore, 20123 Milan, Italy

**Keywords:** cluster analysis, consumer preference, craft beer, neolocalism, specialty beer, sustainability

## Abstract

This study explored beer consumers’ and producers’ perceptions of using local fruit and agroindustrial by-products in brewing. An online survey was conducted in Italy with 496 beer consumers and 54 beer producers. The survey assessed sociodemographic information, consumption behavior, and support for brewery neolocalism, along with brewers’ perceptions of the sustainability of their breweries. Findings showed high consumers’ involvement in sustainable eating and high support for breweries utilizing local resources and operating in a sustainable way. Breweries rated their sustainability practices as moderate, and most of them considered their efforts in using local raw materials and repurposing by-products as “excellent”. Both consumers and producers considered beer made with agroindustrial by-products less tasty than those with local fruit (*p* < 0.05), but no significant difference in willingness to buy was observed (*p* > 0.05). According to cluster analysis results, the ideal consumer profile for sustainable beers was characterized by a higher proportion of females prioritizing sustainability and local sourcing over sensory quality. Therefore, using local materials or agroindustrial by-products can align brewers’ practices with consumers’ preferences, enhancing competitiveness and market positioning by fostering a sense of sustainability and locality.

## 1. Introduction

Sustainability has emerged as an essential concern in the contemporary food and beverage industry, driving changes in consumers’ preferences and production practices [1]. As consumers become more aware of the environmental impacts and cultural heritage, there is a growing demand for products that emphasize local sourcing and sustainable practices [2]. Indeed, the brewing sector, especially in the context of craft beer, provides a clear example of this evolution [3]. Traditional brewing methods usually generate a large amount of waste and require a significant amount of energy, water, and raw materials [4]. As a result, the brewing sector is looking into cutting-edge ways to increase its overall sustainability without sacrificing the quality or appeal of its products to consumers [3]. An example of such sustainable practices is using local ingredients in brewing. Historically, beer production has been associated with industrial-scale processes using global market-sourced malts, hops, and yeast, often leaving consumers unaware of their origins. However, the “Craft Revolution” has changed this perception, emphasizing how deeply rooted beer is in local traditions and landscapes [5,6]. Therefore, promoting the use of local raw materials is crucial for improving sustainability in the brewing sector and enhancing its appeal and value. Indeed, Italy implemented Ministerial Resolution 212/2010 [7], which recognized beer as an agricultural product and defined the so-called “agricultural breweries”. In this context, farmers have to use a percentage of their raw materials that is not less than 51% to produce agricultural beer. This law emphasized the importance that the brewery industry places on local production and sustainability nationally to recognize the value of local sourcing. In general, the term “neolocalism” refers to the importance given to the localization of a brewery and its production [8]. This aspect has gained popularity in the craft beer movement and promotes sourcing and producing locally to strengthen a sense of place and community. This approach supports local economies and boosts the cultural identities of the communities where these breweries are situated [8,9].

In addition, craft beer production has been leading the way in experimenting with sustainable practices through the integration of agroindustrial by-products into the brewing process as a strategy for enhancing sustainability [10]. As reported by da Cunha et al. [11], craft beer production incorporating native fruit and agroindustrial by-products is a subject of interest for consumers seeking new and unique flavors while simultaneously elevating the beer’s nutritional value and environmental benefits by addressing sustainability and circular economy concerns. This strategy of reducing waste could be a signal of the brewery’s commitment to environmental responsibility, thus lowering the brewery’s overall environmental impact and contributing to wider sustainability goals. Fruit pomace and peels (e.g., apple pomace, grape skins, ginger peels) [12,13,14], molasses [15], olive leaves [16], and coffee pulp [17,18] are some examples of by-products that can be used in the brewing process as contributors to unique flavors and aromas, as nutrients to improve yeast health and fermentation performance, as compounds rich in antioxidants, or as prebiotics to develop functional beers [19,20].

Despite this technological progress, few studies have explored consumers’ and producers’ interest in beers brewed with local fruit or agroindustrial by-products [11]. Specifically, this paper aims to fill this gap by exploring, at the same time, the consumers’ and the producers’ attitudes towards two typologies of specialty beers: beer brewed with local fruit—to enhance local identity—and beer brewed with agroindustrial by-products—for circular economy purposes. In so doing, this research follows the examples of recent studies that embraced a holistic approach in investigating the perspectives of all the economic actors to understand the possibility of implementing innovation in markets such as pollinator-friendly [21] or local foods [22]. This comparative approach is particularly effective in identifying the possible alignment between the preferences and instances of these actors so as to assess the potentialities of a new niche market and to identify keywords and strategies to encourage the production of local and sustainable beer, valorizing the craft brewers’ promoters of territorial identity and sustainable innovation. To achieve this general goal, this study suggested providing answers to four research questions related to four specific aims:

RQ1 (consumer-focused question). Are consumers interested in making sustainable food choices, and how does this interest influence their preference for beers from breweries that operate sustainably? The first question was aimed at understanding if consumers are interested in making sustainable choices within their dietary habits and whether they care about the brewing practices of the beer they consume or if they prioritize the sensory quality of the final product.

RQ2 (producer-focused question). How sustainable do the producers view their microbreweries? The second question investigated the perspectives of beer producers regarding their current sustainable practices and the additional actions required to improve sustainability in the brewing industry.

RQ3 (consumer- and producer-focused question). What is the attitude of consumers and producers towards the use of local fruit or agroindustrial by-products in the brewing process? In order to enable a possible match between consumers’ preferences and producers’ offerings, this question evaluates whether the interests of the two key stakeholders in the beer supply chain are aligned or divergent.

RQ4 (consumer-focused question). What consumer profile is most interested in the proposed local and sustainable beer products? The last question was important from a commercialization perspective in order to identify the ideal consumer profile for the proposed sustainable beer types. Indeed, gaining insights into the target consumers’ psychographic and demographic characteristics may help producers tailor their marketing strategies and increase the market acceptance of sustainable beer offerings.

## 2. Materials and Methods

### 2.1. Survey

A questionnaire was developed using Qualtrics^®^ XM platform and administered online via an electronic link distributed from August 2023 to November 2023 in Italy, targeting both beer consumers and producers. A screening question (“Are you a beer producer?”) divided the survey into two sections: the first one consumer-focused and the second one producer-focused. Since producers also consume beer, they were asked to answer both sections since it was also crucial for the study to comprehend the consumption behavior of producers. The estimated time for beer consumers to complete the questionnaire was 12.4 min. For beer producers, it was 15.8 min due to the inclusion of additional questions specific to their brewing and sustainability practices. The selection of the questions for this survey was guided by the research objectives of this study, thus selecting each domain based on prior findings in this field.

#### 2.1.1. Consumer-Focused Section

The consumer-focused section of the questionnaire comprised six sub-sections: (1) frequency of beer and craft beer consumption, based on a scale from “less than once per month” to “more than once per day”, to assess how often participants consume beer and craft beer [23]; (2) involvement in sustainable eating (4 items) rated on a seven-point Likert scale as reported by Van Loo et al. [24], to measure the degree of importance participants place on sustainable eating practices; (3) support for brewery neolocalism questionnaire to measure the participants’ attitude towards local sourcing and sustainability in breweries [9], divided into three domains: (a) local sourcing—3 items—which referred to the support for breweries that use local ingredients in their brewing process or on their pub’s menu; (b) brewery cause activities—4 items—which indicated the support for breweries that operate in a sustainable manner; (c) taste only—2 items—which indicated that the consumer prioritizes the sensory quality of the beer and not how it is produced; all items were evaluated on a seven-point Likert scale; (4) sociodemographic information (gender, age, education level, area of residence, perception of familiar income); (5) attitude towards beer brewed with (a) local fruit and (b) agroindustrial by-products, by using four different seven-point bipolar semantic differential scales (unhealthy/healthy; disgusting/tasty; unsatisfying/satisfying; not interesting/interesting); (6) willingness to buy (WTB) beer made with (a) local fruit and (b) agroindustrial by-products (on a single-item seven-point semantic scale). According to Cela et al. [25], the definition of agroindustrial by-products was provided. The information related to the “involvement in sustainable eating” and “support for brewery neolocalism” domains was collected from both consumers and producers, but only the results from consumers were considered for the statistical analysis of this study.

#### 2.1.2. Producer-Focused Section

Beer producers were asked specific questions to understand their cultural and economic background: company category (micro, small, medium, and large enterprises, according to the EU recommendation 2003/361 [26]) and the founding year of the brewery. Furthermore, producers were asked to evaluate the social, economic, and environmental sustainability of their breweries by using a seven-point scale ranging from “1 = not at all sustainable” to “7 = very sustainable” to rate efforts of breweries for seven different sustainable practices (sourcing of local ingredients, recycling of waste or by-products material, use of renewable energy, recycling or reuse of process water, use of recyclable packing, use of reusable glass bottles or containers, recovery of CO_2_) by using a five-point scale ranging from “1 = very poor” to “5 = excellent” [27], in order to identify areas needing improvement to enhance overall sustainability [28].

### 2.2. Participants

The survey was shared mainly among students and staff of the University of Gastronomic Sciences, all the partners of the NODES project within which this activity was included (https://www.ecs-nodes.eu/en/7-secondary-agroindustry, last accessed on 16 July 2024), and via social media platforms. Additionally, industry associations within the brewing sector were engaged, and the survey compilation was promoted by participating in relevant beer conferences to reach a broader audience of beer producers and consumers. Participants received an introductory statement explaining the study’s purpose and ensuring data anonymity. Participation in this study was voluntary. Informed consent was obtained from participants before collecting responses. To ensure the quality of the data [29], some responses were excluded due to incomplete answers or a lack of informed consent. Following data validation and the exclusion of incomplete responses, 496 valid questionnaires from consumers and 54 from producers were considered for statistical analysis. This study was conducted in compliance with the Declaration of Helsinki. Approval for the research protocol was obtained from the Ethics Committee of the University of Gastronomic Sciences (Ethics Committee Proceedings n. 2023.03).

### 2.3. Statistical Analysis

The statistical analyses performed on data from consumers and producers are reported in the following two separate sections. Descriptive analysis was used to analyze the questionnaire results. Categorical variables were presented as frequencies, while numerical variables were reported as mean ± standard deviation. All statistical analyses were performed using XLSTAT Premium software (Version 2023.3.0, Addinsoft, Paris, France).

#### 2.3.1. Consumers-Focused Section

The results from the questionnaires “involvement in sustainable eating” and “support for brewery neolocalism” were first analyzed using reliability analysis. Cronbach’s α coefficients, computed to individuate the internal consistency, were considered reliable for values > 0.6 [30,31].

A one-way ANOVA, followed by Tukey’s post hoc test (α = 0.05), was performed in order to identify differences in mean scores of the “support for brewery neolocalism” questionnaire domains. Pearson correlation analysis was performed to identify significant correlations between the domains “involvement in sustainable eating” and “support for brewery neolocalism”. Moreover, cluster analysis was performed to classify consumers according to their preference patterns, particularly based on “involvement in sustainable eating” and “support for brewery neo-localism.” The optimal number of clusters was determined using the Agglomerative Hierarchical Clustering (AHC) technique based on Ward’s agglomerative method. Specifically, the Hartigan index was employed as a criterion to assess the quality of the clustering solution by comparing the within-cluster sum of squares across different numbers of clusters [32,33]. Then, k-means cluster analysis was applied, and clusters were evaluated for high internal homogeneity (within-cluster) and high external heterogeneity (between-cluster). Independent two-sample t-tests were performed to individuate differences between clusters in the “involvement in sustainable eating” and “support for brewery neolocalism” domain values and between and within clusters in terms of attitudes towards both specialty beers. A chi-square analysis was performed to compare clusters based on their sociodemographic characteristics. 

#### 2.3.2. Producers-Focused Section

A one-way ANOVA, followed by Tukey’s post hoc test (α = 0.05), was performed to identify differences in ratings of the efforts of breweries for sustainable practices. Independent two-sample *t*-tests were performed to identify differences in attitudes towards beer brewed with local fruit and agroindustrial by-products according to the foundation year of breweries.

#### 2.3.3. Consumers and Producers-Focused Section

Independent two-sample *t*-tests were performed to individuate differences between attitudes towards and willingness to buy beer brewed with local fruit and agroindustrial by-products, both for consumers and producers.

## 3. Results and Discussion

### 3.1. Sociodemographic

**Figure 1:**
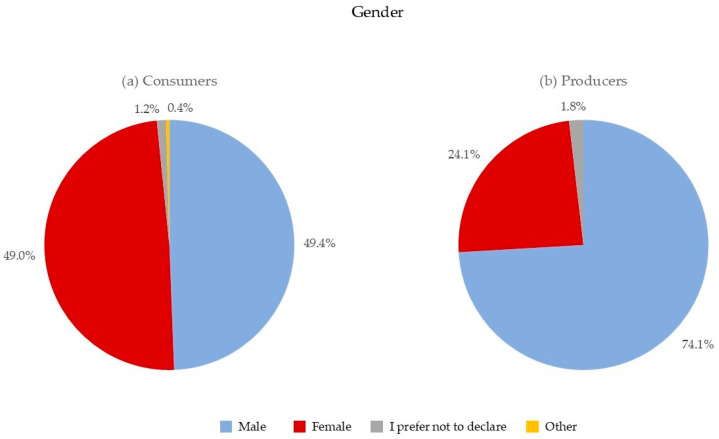
Gender distribution for (**a**) consumers and (**b**) producers.

Regarding sociodemographic profiles, among the 496 consumers, 49.0% were females, 49.4% were males, 1.2% preferred not to disclose their gender, and 0.4% identified themselves as belonging to other genders (Figure 1a). Therefore, for consumers, there was a balanced representation of two main genders. Conversely, considering only the producers (*n* = 54), 74.1% were males, 24.1% were females, and 1.8% preferred not to disclose their gender (Figure 1b). This gender disparity among producers aligns with trends in the Italian brewing industry, where women are underrepresented in leadership roles within breweries, a sector traditionally dominated by men [34].

**Figure 2:**
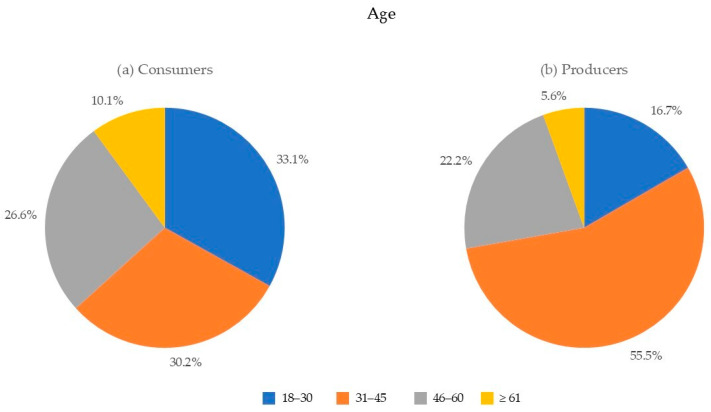
Age distribution for (**a**) consumers and (**b**) producers.

Most producers and consumers were under 45 years old (72.2% and 63.3%, respectively), suggesting a younger demographic actively involved in the craft beer market (Figure 2).

**Figure 3:**
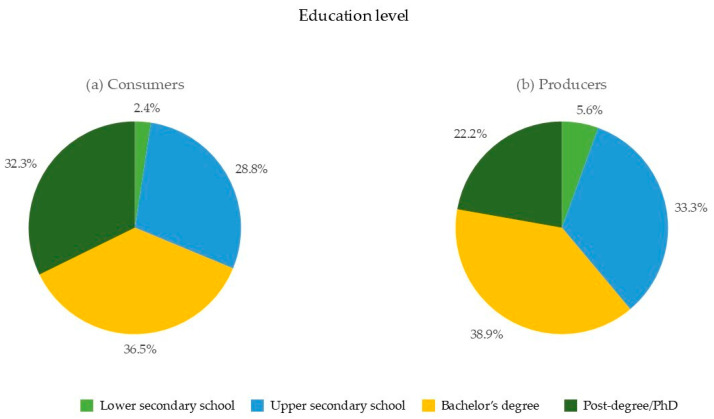
Education level of (**a**) consumers and (**b**) producers.

Regarding educational background, a substantial proportion of respondents had completed at least a bachelor’s degree (68.8% consumers and 61.1% producers), indicating a highly educated participant base. Furthermore, a high school diploma was held by 28.8% of consumers and 33.3% of producers, whereas 2.4% of consumers and 5.6% of producers reported having completed lower secondary school as the highest educational level (Figure 3).

**Figure 4:**
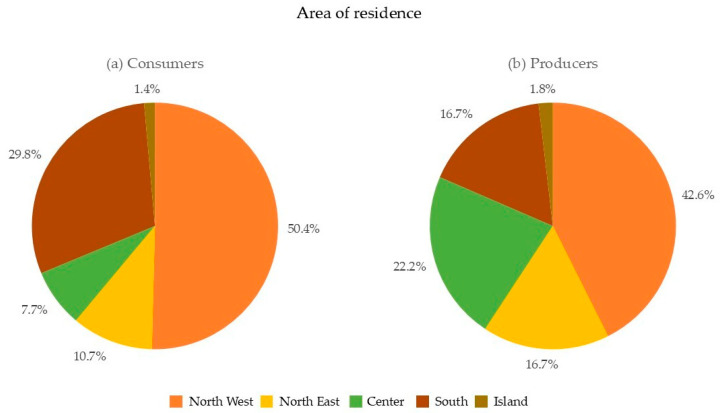
Area of residence of (**a**) consumers and (**b**) producers.

As reported in Figure 4, Northern Italy accounted for the majority of responses (61.1% consumers and 59.3% producers), in line with the region’s higher concentration of craft breweries and beer drinkers, as reported by AssoBirra [35]. On the other hand, 7.7% of consumers and 22.2% of producers were from the central regions of Italy. Additionally, 31.2% and 18.5% of consumer and producer respondents, respectively, originated from the islands and southern Italy.

**Figure 5:**
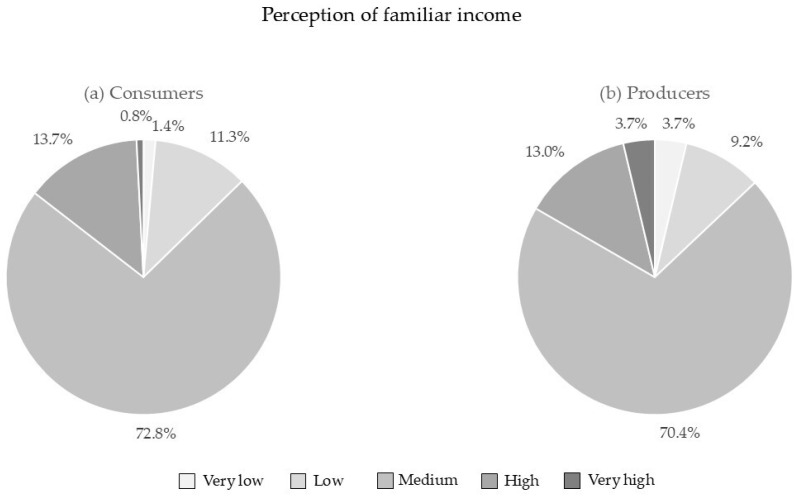
Perception of familiar income by (**a**) consumers and (**b**) producers.

Moreover, most consumers and producers (72.8% and 70.4%, respectively) reported having a medium household income, whereas 12.7% and 13.0%, respectively, thought they had a very low/low household income, compared to 14.5% of consumers and 16.6% of producers who considered having a high/very high household income (Figure 5).

Finally, 50 out of 54 breweries that responded to the questionnaire (92.6%) were micro-sized enterprises (turnover < EUR 2 million), whereas 3.7% (n = 2), 1.8% (n = 1), and 1.9% (n = 1) of producers were small (turnover ≤ EUR 10 million), medium (turnover ≤ EUR 50 million), and big-sized enterprises (turnover > EUR 50 million), respectively.

### 3.2. Consumers’ Interest in Sustainable Food Choices and Support for Brewery Neolocalism

Exploring the consumers’ interest in sustainable food choices is important to understand their social and environmental commitment, thus guiding the producers in aligning their practices with consumers’ values [36]. To address the first research question, the consumers’ interest in sustainable choices was assessed by examining their involvement in sustainable eating, as reported by Van Loo et al. [24]. Consumers demonstrated a high level of involvement in sustainable eating, with a mean domain score of 5.7 ± 1.1 on a seven-point Likert scale and Cronbach’s α = 0.917, highlighting a high internal consistency (Table 1).

**Figure 6:**
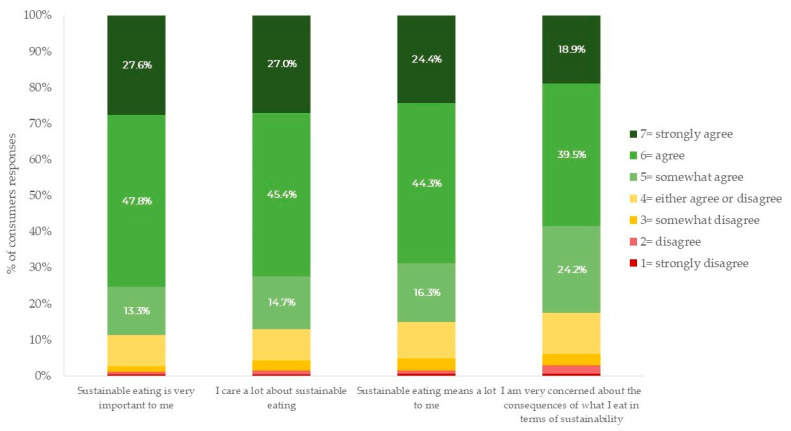
Consumers’ responses to the “involvement in sustainable eating” questionnaire on a seven-point Likert scale (1 = strongly disagree; 7 = strongly agree).

As shown in Figure 6, by considering the percentage of consumers who showed agreement with the items of the domain (scores above the midpoint of the seven-point Likert scale), a significant majority (88.7%) of consumers’ respondents agree that eating sustainably is very important to them. Similarly, 87.1% stated that they care a lot about sustainable eating, while 85.0% of consumers reported that sustainable eating means a lot to them. Moreover, about 82.6% of consumers expressed high concern about the sustainability consequences of what they ate. This finding indicated a strong consumer inclination towards sustainable products, suggesting that to appeal to this environmentally conscious market, producers would be advantageous by implementing and emphasizing sustainable production techniques.

**Figure 7:**
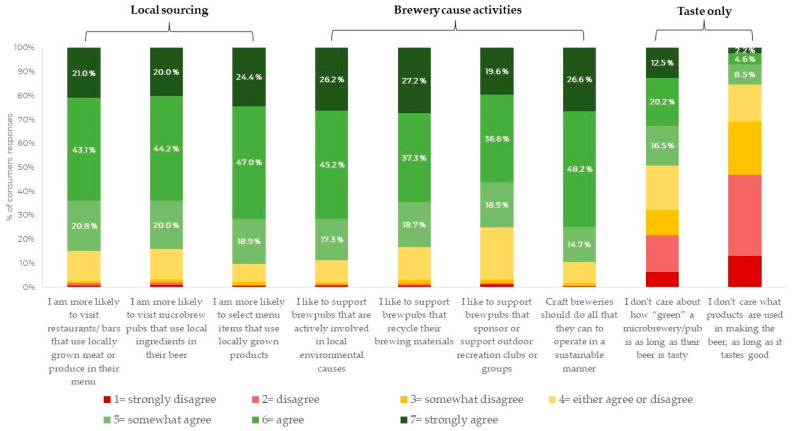
Consumers’ responses to the “support for brewery neolocalism” questionnaire on a seven-point Likert scale (1 = strongly disagree; 7 = strongly agree).

Understanding the extent to which consumers prioritize sustainability in their daily lives as well as in other realms, such as beverage choices, was among the specific objectives of this study. The results of the questionnaire applied to evaluate the degree of support for brewery neolocalism are reported in Table 2. Consumers showed solid support for breweries utilizing local resources and raw materials with a mean “local sourcing” domain score of 5.7 ± 1.1 (Cronbach’s α = 0.833). Indeed, considering the percentage of consumers who selected scores above the midpoint of the seven-point Likert scale (Figure 7), 84.9% of consumers were more likely to visit restaurants/bars using locally grown meat or produce, 84.2% preferred microbrew pubs using local ingredients, and 90.3% reported being inclined to select menu items featuring locally grown products. Additionally, there was significant support for breweries that engage in sustainable practices, with a mean “brewery cause activities” domain score of 5.7 ± 1.1 (Cronbach’s α = 0.815). A significant proportion of consumers (88.7%) showed high support for brewpubs involved in local environmental causes; brewpubs that recycle their brewing materials were supported by 83.2% of consumer respondents; supporting outdoor recreation clubs or groups was considered important to 75.1% of consumers; and 89.5% of consumers agreed that craft breweries should do all that they can to operate in a sustainable manner. The “taste only” domain obtained an average score of 3.6 ± 1.6. This domain, while showing acceptable internal consistency (Cronbach’s α = 0.645), presented a greater degree of variation in responses. Only 49.2% of consumers (who showed agreement with the items by selecting the scores above the midpoint of the seven-point Likert scale) did not care about how “green” a microbrewery/pub is if the beer is tasty. A smaller percentage of consumers (15.3%) did not care about what products were used in making the beer as long as it tasted good.

According to one-way ANOVA results, the mean scores of the “local sourcing” and “brewery cause activities” domains were significantly higher than the mean score for the “taste only” domain (*p* < 0.05), as reported in Table 2. This discrepancy confirmed that beer consumers give greater significance to extrinsic attributes such as sustainability and local sourcing than only to intrinsic characteristics (particularly taste) [37]. Indeed, these findings aligned with recent literature that highlights the increasing significance of sustainability in consumer decision-making across different food and beverage sectors [38] as a result of growing awareness about the ecological and social impacts of food production and consumption [39]. This trend is particularly evident in the market segments for organic foods [40,41,42], plant-based alternatives [43,44,45], and eco-friendly packaged products [46,47,48], highlighting a significant positive impact of sustainability features on consumers’ acceptability and the pivotal role of ethical issues in driving a shift towards more sustainable consumption patterns. Therefore, in the context of this market trend, this study extended these insights to the beer industry, which is not usually closely associated with sustainability, thus encouraging brewers to innovate and adopt sustainable practices to meet consumers’ preferences.

The results of the Pearson correlation analysis conducted to understand how consumers’ interest in sustainable food choices relates to their interest in sustainable practices within the brewing industry are reported in Table 3. The domain “involvement in sustainable eating” was significantly (*p* < 0.05) and positively correlated with both the “local sourcing” (*r* = 0.413) and “brewery cause activities” (*r* = 0.527) domains. Conversely, there was a significant negative correlation between the “involvement in sustainable eating” domain and the “taste only” domain (*r* = −0.344, *p* < 0.05). These correlations highlighted how important sustainability is to consumers and showed that people who follow sustainable eating are more likely to support breweries that use sustainable practices and local sourcing.

Therefore, these results illustrated a significant shift in consumer behavior, where sustainability and localism are becoming pivotal factors in the decision-making process for beer choice. For producers, this could imply that marketing strategies should not only focus on the sensory appeal of their products but also on their sustainable practices and local contributions. The general public’s growing awareness of environmental issues and interest in supporting local economies are probably the main drivers of this trend. Although it can be challenging for producers to find a balance between sustainable production and sensory quality, there is a strong incentive to invest in these areas, given the clear demand for sustainability from consumers [49].

### 3.3. Producers’ Perception of Degree of Sustainability

In this study, most of the breweries that responded to the questionnaire were micro-sized enterprises. This economic background aligned with the broader landscape of craft breweries in Italy, as documented by the Union Birrai report [50]. The predominance of micro-sized enterprises among the respondents enhanced the representativeness of the sample of this study, as it reflected the typical economic scale of craft breweries in Italy. These results could be relevant in the context of sustainability. Indeed, compared to industrial breweries, microbreweries may be more willing to implement sustainable practices driven by operational flexibility and the producers’ values that emphasize boosting local identity and economy [5]. The majority of breweries in this study (55%) were founded after the implementation of the Italian Craft Beer Law, promulgated in 2016 [51], which played a significant role in the expansion of the sector. The results from the information about the foundation year of the breweries involved in this study highlighted the helpful effect of legislative support on craft industry growth [52]. 

The adoption of sustainable practices is crucial for breweries aiming to minimize their environmental impact and enhance their social responsibility. Key sustainable actions include reducing water consumption, using renewable energy sources (i.e., photovoltaic, solar thermal, and geothermal), and repurposing by-products [53,54]. To respond to the second research question, this study looked into how beer producers thought about their breweries’ sustainability across various practices, identifying areas of strength and those needing additional efforts.

Findings from producers’ responses (n = 54) showed that they perceive their brewing processes as moderately sustainable, with an average score of 4.5 ± 1.6 on a seven-point scale. This moderate rating suggested that producers realize the progress that has been made in the direction of sustainability, but they also recognize the need for further improvement.

**Figure 8:**
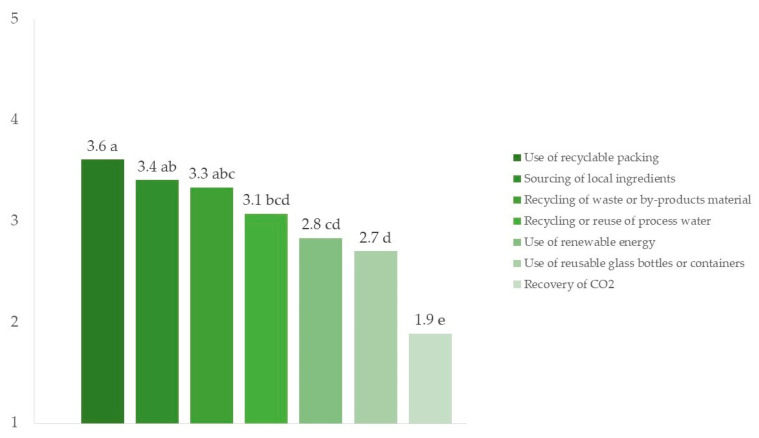
Ratings of sustainable practices according to producers’ responses (n = 54). (1 = “very poor”, 2 = “poor”, 3 = “fair”, 4 = “good”, 5 = “excellent”). Different letters indicate significantly different mean values (*p* < 0.05).

Producers identified several sustainable practices in which they thought their breweries operated effectively. According to the ANOVA results (Figure 8), the use of recyclable packaging received the highest ratings, indicating that this practice is well-established. Following this, sourcing local ingredients and recycling waste or by-product materials were also rated positively. Indeed, these practices were not significantly different from the use of recyclable packaging (*p* > 0.05), thus still being considered among the most prioritized sustainable practices. These actions reduce environmental impact and improve interactions between communities and the local economy. Recycling or reuse of processed water was found to be statistically less applied than the most widely adopted sustainable practice (use of recyclable packaging) but not statistically different from the mean ratings of the second and third most popular sustainable practices (sourcing of local ingredients and recycling waste or by-product materials). Other practices that did not receive ratings above “fair” (mean rating above 3 out of 5) included the use of renewable energy and the use of reusable glass bottles or containers. Moreover, the recovery of CO_2_ was considered the least efficient practice, with a mean rating statistically different (*p* < 0.05) from all other practices. This highlighted a critical area for improvement, as effective CO_2_ recovery can significantly reduce greenhouse gas emissions. Findings from this study could serve as suggestions for producers to identify focal points for upcoming initiatives related to sustainability.

**Figure 9:**
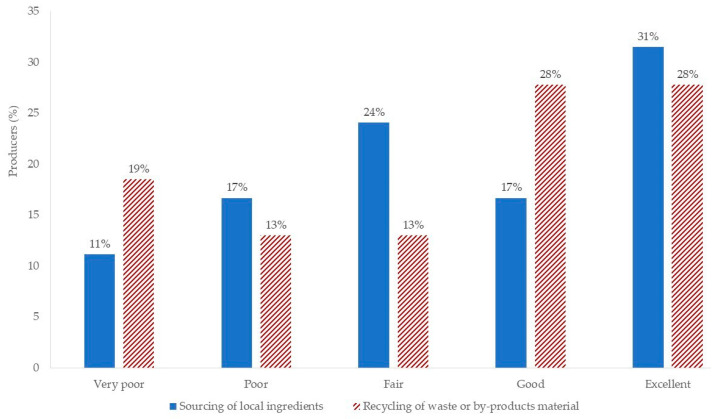
Ratings of the efforts of breweries for two sustainable practices: sourcing of local ingredients and recycling of waste or by-product material.

Figure 9 illustrates producers’ ratings for two key sustainable practices: sourcing local ingredients and recycling waste or by-product materials. Notably, 31% and 28% of producers rated their efforts in these areas as “excellent.” This indicated that producers were very interested in and involved with these practices, aligning with the larger industry trend toward sustainability [4]. On average, beer producers rated their breweries as “fair” for both practices, suggesting further development and enhancement potential. These findings offered insightful information about the sustainability landscape of the Italian craft beer market. Recognizing the strengths and weaknesses of breweries can help policymakers, entrepreneurs, and breweries themselves create targeted plans for improving sustainability. However, producers’ education and awareness about the environmental and social benefits of sustainable practices are crucial, as they extend beyond merely increasing market appeal and meeting emerging market trends.

### 3.4. Attitude towards Beer Brewed with Local Fruit or Agroindustrial By-Products

Consumers’ appreciation of the taste, price, and overall quality of beer should be balanced with the sustainable features of the beer. Furthermore, it is essential for producers that the financial viability of the whole process is not compromised by the practical challenges associated with sustainable practices. Thus, matching the interests of consumers and producers is pivotal for the broad implementation of sustainable brewing practices. Therefore, the third research question of this study aimed at investigating the attitude of both beer consumers and producers towards two proposed specialty beers: beer brewed with (a) local fruit and (b) agroindustrial by-products.

**Figure 10:**
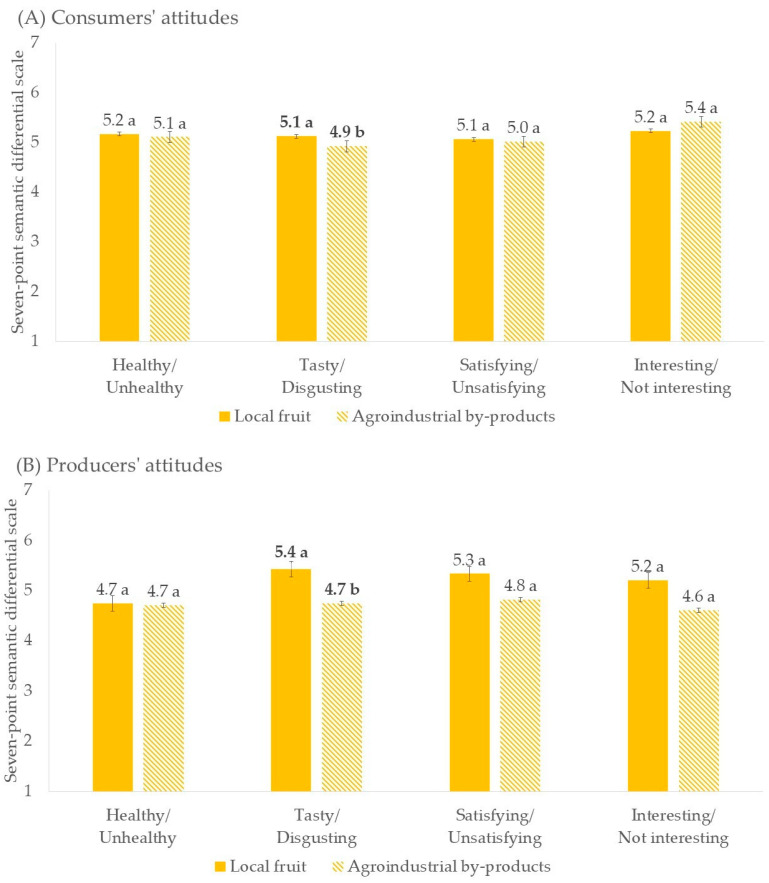
Consumers’ (**A**) and producers’ (**B**) attitudes towards beer brewed with local fruit and with agroindustrial by-products. Different letters indicate significantly different values between the two samples (*p* < 0.05).

The analysis of consumers’ and producers’ attitudes revealed generally positive perceptions (Figure 10). All scores were above the midpoint of the scale (>4 on a seven-point scale), suggesting that both consumers and producers showed a positive attitude towards the incorporation of both local fruit and agroindustrial by-products in the brewing process, thus recognizing the market potential of these specialty beers. Consumers (Figure 10A) rated high scores on all four attributes (healthy, tasty, satisfying, and interesting) for both beer brewed with local fruit and beer brewed with agroindustrial by-products. Beer producers (Figure 10B) also demonstrated a favorable attitude towards these specialty beers. 

However, independent two-sample t-test results (Figure 10) showed that both consumers and producers considered beer brewed with agroindustrial by-products significantly less tasty than those produced with local fruit (*p* < 0.05). This finding aligned with existing literature, which indicates that by-products are often perceived as lower in quality compared to conventional food products [55]. The sensory appeal, particularly taste, plays a crucial role in consumer acceptance [56]. Therefore, the perception of reduced taste quality could be a barrier to the acceptability of beer made with agroindustrial by-products, which can be perceived negatively by consumers who prefer traditional beer flavors.

In addition, to understand how the Italian Craft Beer Law, promulgated in 2016, has influenced the brewers’ attitudes and production methods, independent two-sample *t*-tests were conducted to investigate differences in attitudes towards the two specialty beers between breweries established before (n = 24) and after (n = 30) 2016. As shown in Table 4, beers brewed with local fruit were considered significantly more satisfying (*p* < 0.05) by newer breweries (foundation year after 2016) than breweries founded before the Italian Craft Beer Law. Conversely, no significant differences were found for the other evaluated attitudes (*p* > 0.05). Overall, the data suggested that the principles of craft brewing, particularly regarding sustainability and innovation, were already well-established among Italian breweries before the implementation of the 2016 law. Indeed, the craft brewers were already aligned with sustainable principles and created a cultural movement later codified by the 2016 law [5], making the legislative specification an official declaration of the already existing practices rather than a stimulus for new ones.

Despite the difference in taste perception, results from independent two-sample *t*-tests highlighted no significant difference in willingness to buy between the two types of beers for both consumers and producers (*p* > 0.05). On a seven-point scale, consumers rated WTB beer made with local fruit and agroindustrial by-products at 5.5 ± 1.5 and 5.6 ± 1.4, respectively. Producers provided comparable mean scores: 5.7 ± 1.4 and 5.3 ± 1.5 for beer brewed with local fruit and with agroindustrial by-products, respectively. This suggested that both groups find these beers appealing due to their sustainability and local sourcing, even though they may have unfamiliar sensory characteristics. Therefore, these locally sourced and sustainable beer options may encourage neolocalism and sustainable brewing methods. Moreover, findings from this study suggested that there may be a market for beers made using environmentally friendly methods due to the high willingness to buy.

However, the perceived negative impact of agroindustrial by-products on beer taste was the major concern, despite the general positive attitude. Thus, it is crucial to optimize the brewing process to use these unconventional and eco-friendly ingredients without sacrificing the flavor profile or the overall sensory quality. This optimization may involve choosing the proper by-products with a minimal impact on flavor or creating novel brewing methods that enhance the flavor characteristics of beers made using by-products.

### 3.5. Consumer Segmentation

To determine the most appropriate cluster of consumers and give producers insights into the ideal consumer profile for accepting innovative beer offerings (answering the fourth research question of this study), the segmentation of consumers according to their preference patterns was crucial. Producers could better meet the preferences and expectations of targeted consumer segments by customizing their marketing strategies and product development efforts based on their understanding of distinct consumer profiles. This strategy boosts the efficacy of promotional efforts and increases the possibility of a successful market for these new beer offerings [57].

**Figure 11:**
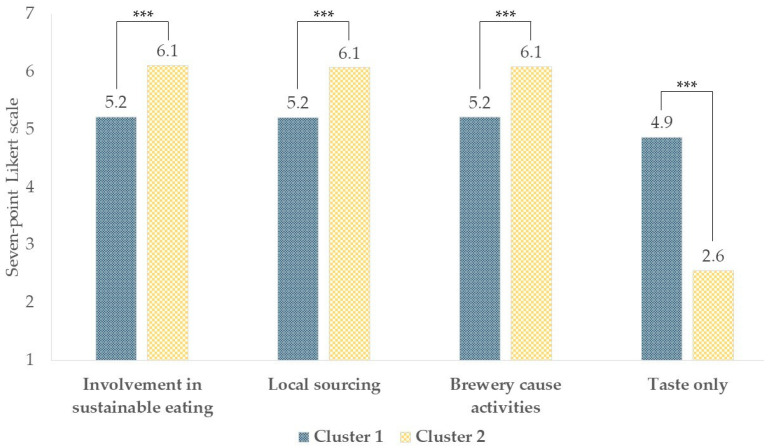
Differences between “hedonic consumers” (Cluster 1; n = 214) and “sustainable consumers” (Cluster 2; n = 251) in “involvement in sustainable eating” and “support for brewery neolocalism” domains. *** *p* < 0.001 according to independent two-sample *t*-tests.

The Hartigan index, resulting from AHC analysis, indicated that the optimal number of consumer clusters was two. K-means clustering by the Euclidean distance method was subsequently carried out, specifying a two-cluster solution to segment consumers according to their involvement in sustainable eating and support for brewery neolocalism. Consumers with negative silhouette scores (n = 31) were excluded from further analysis, resulting in Cluster 1 (n = 214) and Cluster 2 (n = 251). Based on findings from t-tests performed to individuate differences between clusters in terms of “involvement in sustainable eating” and “support for brewery neolocalism”, it is possible to state that the most discriminating factor between the two consumer clusters was the priority they placed on the taste of the beer (Figure 11). Indeed, Cluster 1 was defined as “hedonic consumers” since they showed statistically lower mean values for the “involvement in sustainable eating”, “local sourcing”, and “brewery cause activities” domains (5.2 ± 1.1, 5.2 ± 0.9, and 5.2 ± 0.9, respectively) and higher mean values for the “taste only” domain (4.9 ± 0.9) compared to Cluster 2 (*p* < 0.0001), suggesting that Cluster 1 was more interested in the sensory aspect of the final beer, which in general is considered one of the most important drivers for beer choice [56]. Nevertheless, Cluster 1 showed a generally favorable attitude towards sustainability as it provided mean scores above the midpoint of the seven-point Likert scale (value > 4 for “involvement in sustainable eating”, “local sourcing”, and “brewery cause activities”). However, when considering the “taste only” domain, the difference between the two clusters was more pronounced. Opposed to Cluster 1, Cluster 2 included consumers with statistically higher values of “involvement in sustainable eating”, “local sourcing”, and “brewery cause activities” (6.1 ± 0.7, 6.1 ± 0.8, and 6.1 ± 0.7, respectively) and lower values of the “taste only” domain, thus defining “sustainable consumers” (*p* < 0.0001). Indeed, Cluster 2 provided a statistically lower value to the “taste only” domain compared to Cluster 1 (*p* < 0.0001), and the score was below the midpoint of the seven-point scale (2.6 ± 0.8). 

**Figure 12:**
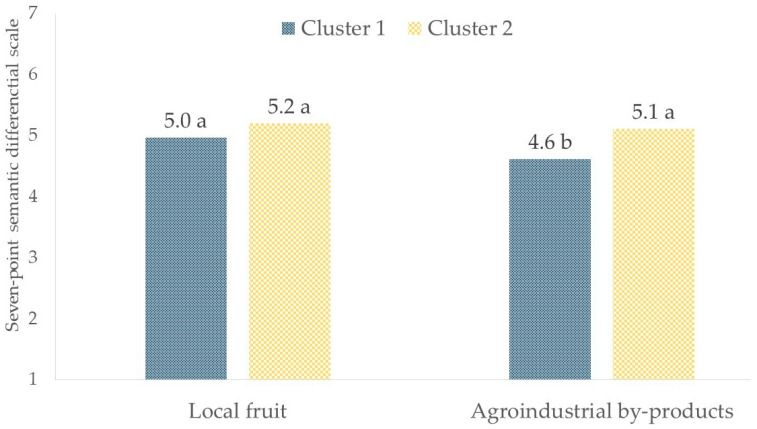
Difference in “tasty” attitude between clusters. Different letters indicate significantly different values between the clusters (*p* < 0.05).

According to an independent two-sample t-test, significant results were found when exploring the differences between clusters regarding the “tasty” attribute and the attitude of each cluster towards these proposed specialty beers. A significant difference between clusters emerged for the beer brewed with agroindustrial by-products (*p* < 0.05), with Cluster 1 giving it a lower rating than Cluster 2 (Figure 12). On the other hand, for beer brewed with local fruit, there was no significant difference (*p* > 0.05) in the “tasty” ratings between the two clusters (Figure 12).

**Figure 13:**
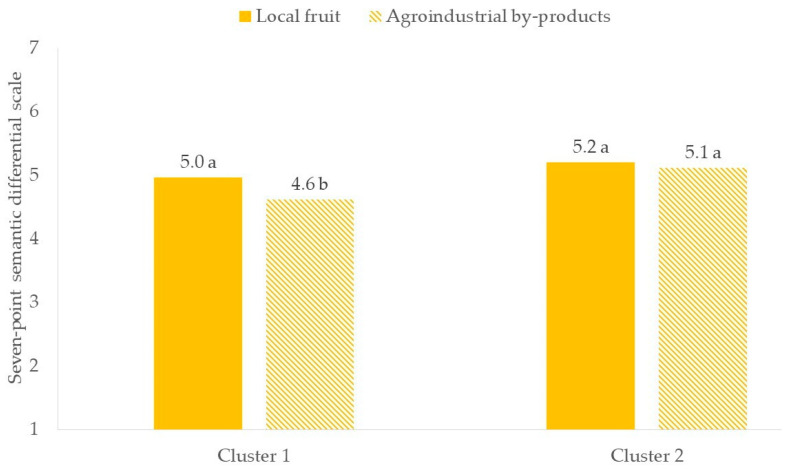
Differences in “tasty” attitudes within clusters. Different letters indicate significantly different values between the two specialty beers (*p* < 0.05).

Within the clusters, a significant difference was observed only within Cluster 1 since it included “hedonic consumers” (Figure 13). Indeed, this group rated the beer produced with agroindustrial by-products as significantly less tasty compared to the beer brewed with local fruit (*p* < 0.05), showing a clear preference for beer with more familiar flavors and more closely aligned with traditional production methods, such as those brewed with local fruit. On the other hand, beer made with agroindustrial by-products may introduce unusual flavors that, although innovative, may not immediately attract consumers who give priority to taste above all else. This might be the result of a lack of familiarity or a high level of food neophobia [58,59]. This suggested that, even though consumers were open to sustainable practices, their primary concern was still the sensory quality of the product. For brewers, these findings suggested the need for careful formulation in order to meet the different demands of various consumers’ segments.

Therefore, each cluster was characterized for sociodemographic information to provide information to the business companies and suggest guidelines to individuate the proper consumer profile to promote and share this new type of beer. Chi-square test results showed a significant difference between clusters by gender (*p* < 0.01) but not by the other sociodemographic variables or beer consumption behavior (Table 5). In particular, the “sustainable consumers” cluster (Cluster 2) was characterized by the highest proportion of female participants. This result confirmed the differences in beer choice between males and females, as also stated in previous studies [56,60], suggesting that female consumers could be an ideal consumer profile more prone to trying local and sustainable beer offerings.

### 3.6. Limitations

In terms of limitations, this study focused on the craft beer segment, a more innovative fraction of a market dominated by industrial production [61]. In this respect, the general attitude toward sustainability of the interviewed consumers cannot be projected to the entirety of the sector. However, considering how the craft beer sector played a forerunner in most of the innovations later adopted by industrial producers (not-filtered, fruity beer, etc.) [62], the new developments that will occur in this sector may be later transferred to the beer market at large. On the other hand, the attitude of industrial brewers was not analyzed in this study and remains to be investigated.

## 4. Conclusions and Future Perspectives

This study aimed to understand the alignment of the production practices of craft breweries with consumers’ preferences for sustainability and neolocalism. Findings indicated a strong consumer inclination towards sustainable practices and local sourcing within the Italian craft beer market. The results showed that consumers’ purchase decisions for beer were significantly influenced by their desire for sustainability and neolocalism in beer production, suggesting the growing importance of these characteristics in the contemporary craft beer market. 

From the producers’ perspective, this study revealed a moderate degree of perceived sustainability in their brewing processes. While improvements in areas such as waste recycling, the use of local ingredients, and recyclable packaging were acknowledged, significant advances are still required in terms of CO_2_ recovery and the use of renewable energy. These findings gave breweries a roadmap for improving their sustainability initiatives, aligning with consumer expectations, and contributing to a more sustainable industry.

Both consumers and producers were found to be open to sustainable beer options when attitudes towards beer brewed with local fruit and agroindustrial by-products were investigated. Despite concerns about the taste of beers made with agroindustrial by-products, the overall willingness to buy these beers indicated a market potential driven by their environmental benefits. Optimizing the brewing process to improve the sensory aspects of these beers could further enhance their market appeal, as could ad hoc communication campaigns aimed at making visible and readable to consumers the positive environmental externalities of these productions.

Two distinct consumer clusters were identified through consumer segmentation: “hedonic consumers”, who prioritized sensory quality, and “sustainable consumers”, who emphasized sustainability and local sourcing. The latter cluster, characterized by a higher proportion of female participants, was considered a key target demographic for marketing local and sustainable beers. Therefore, producers should focus their efforts on the market segment characterized by a higher level of involvement in sustainable eating and strong support for brewery neolocalism, representing the ideal consumer profile for accepting innovative, sustainable beer offerings. To appeal to these consumers, marketing strategies should highlight sustainability features and use locally sourced ingredients. By focusing on optimizing the brewing process and educating consumers, the craft beer industry can successfully innovate in a way that meets both sustainability goals and consumers’ expectations. These efforts could enhance the marketability of such beers and contribute to broader environmental, social, and economic sustainability within the brewing industry.

Future research should continue exploring innovative brewing techniques and other factors and motivations behind consumers’ choice of sustainable beer offerings to further support adopting sustainable practices in the craft beer sector. Additionally, further studies should investigate the usage and waste of electricity in the brewing industry since this is an energy-intensive sector [4]. Indeed, investigating waste management practices, energy efficiency measures, and the adoption of renewable energy sources may help reduce the environmental impact of the brewing process. Moreover, additional studies should explore the legal framework behind brewing with unconventional ingredients (i.e., agroindustrial by-products) to ensure that these novel methods comply with national beer regulations and meet food safety standards. In conclusion, beyond the environmental advantages, brewing more sustainable beers may also have an effect on society, driving consumers’ behavior and attitudes towards more sustainable consumption habits. Sustainable brewing techniques could attract an increasing number of eco-conscious consumers who place significant importance on sustainability in their purchasing decisions.

## Figures and Tables

**Table 1:** “Involvement in sustainable eating” questionnaire results.

Domain	Item	Mean Item (SD)	Mean Domain (SD)	Cronbach’s α
Involvement in sustainable eating	Sustainable eating is very important to me	5.9 (1.0)	5.7 (1.1)	0.917
	I care a lot about sustainable eating	5.8 (1.1)		
	Sustainable eating means a lot to me	5.7 (1.1)		
	I am very concerned about the consequences of what I eat in terms of sustainability	5.5 (1.2)		

**Table 2:** “Support for brewery neolocalism” questionnaire results.

Domain	Item	Mean Item (SD)	Mean Domain (SD)	Cronbach’s α
Local sourcing	I am more likely to visit restaurants/bars that use locally grown meat or produce in their menu	5.7 (1.1)	5.7 (1.1) ^a^	0.833
	I am more likely to visit microbrew pubs that use local ingredients in their beer	5.6 (1.1)		
	I am more likely to select menu items that use locally grown-products	5.8 (1.0)		
Brewery cause activities	I like to support brewpubs that are actively involved in local environmental causes	5.8 (1.0)	5.7 (1.1) ^a^	0.815
	I like to support brewpubs that recycle their brewing materials	5.7 (1.2)		
	I like to support brewpubs that sponsor or support outdoor recreation clubs or groups	5.5 (1.2)		
	Craft breweries should do all that they can to operate in a sustainable manner	5.9 (1.0)		
Taste only	I do not care about how “green” a microbrewery/pub is as long as their beer is tasty	4.3 (1.8)	3.6 (1.6) ^b^	0.645
	I do not care what products are used in making the beer as long as it tastes good	3.0 (1.5)		

Different letters indicate statistically significant differences according to Tukey’s post-hoc test.

**Table 3:** Correlation between “involvement in sustainable eating” and “support for brewery neo-localism” domains.

Domain	Involvement in Sustainable Eating	Local Sourcing	Brewery Cause Activities
Local sourcing	0.413 ***		
Brewery cause activities	0.527 ***	0.586 ***	
Taste only	−0.344 ***	−0.319 ***	−0.378 ***

*** significant correlation at *p* < 0.001.

**Table 4:** Differences in attitudes towards beer brewed with local fruit and agroindustrial by-products between breweries founded before (n = 24) and after (n = 30) the Italian Craft Beer Law.

Attitudes	Foundation Year of Brewery	Local Fruit		Agroindustrial By-Products	
		Mean (SD)	*p*-Value *	Mean (SD)	*p*-Value *
Healthy/Unhealthy	Before the Italian Craft Beer Law	4.3 (1.8)	0.125	4.5 (1.7)	0.421
	After the Italian Craft Beer Law	5.1 (1.7)		4.9 (1.6)	
Tasty/Disgusting	Before the Italian Craft Beer Law	5.1 (1.9)	0.191	4.6 (1.8)	0.536
	After the Italian Craft Beer Law	5.7 (1.5)		4.9 (1.5)	
Satisfying/Unsatisfying	Before the Italian Craft Beer Law	4.8 (1.8)	0.048	4.8 (2.0)	0.811
	After the Italian Craft Beer Law	5.7 (1.5)		4.9 (1.6)	
Interesting/Not interesting	Before the Italian Craft Beer Law	4.6 (2.2)	0.058	4.6 (2.1)	0.965
	After the Italian Craft Beer Law	5.7 (1.7)		4.6 (2.0)	

* *p*-value < 0.05 = statistically significant difference according to independent two-sample *t*-test.

**Table 5:** Sociodemographic differences between clusters.

Sociodemographic Variable	Cluster 1 (n = 214)		Cluster 2 (n = 251)		Pearson Chi-Square	*p*-Value
	*N*	%	*N*	%		
*Gender*					11.896	0.008
Female	87	40.65	140	56.00		
Male	124	57.94	105	42.00		
I prefer not to declare	2	0.93	4	1.60		
Other	1	0.47	1	0.40		
*Age*					2.233	0.526
18–30	72	33.64	87	34.80		
31–45	67	31.31	69	27.60		
46–60	53	24.77	74	29.60		
>61	22	10.28	20	8.00		
*Education level*					3.653	0.301
Lower secondary school	6	2.80	5	2.00		
Upper secondary school	60	28.04	78	31.20		
Bachelor’s degree	71	33.18	96	38.40		
Post-degree/PhD	77	35.98	71	28.40		
*Area of residence*					4.086	0.394
North West	114	53.27	119	47.60		
North East	23	10.75	29	11.60		
Center	15	7.01	18	7.20		
South	57	26.64	82	32.80		
Island	5	2.34	2	0.80		
*Perception of familiar income*					7.547	0.110
Very low	6	2.80	1	0.40		
Low	21	9.81	32	12.80		
Medium	158	73.83	178	71.20		
High	26	12.15	38	15.20		
Very high	3	1.40	1	0.40		
*Frequency of beer consumption*					9.694	0.138
Less than once per month	19	8.88	34	13.60		
Once per month	13	6.07	24	9.60		
Less than once per week	26	12.15	36	14.40		
Once per week	57	26.64	63	25.20		
2–4 times per week	74	34.58	74	29.60		
Once per day	16	7.48	16	6.40		
More than once per day	9	4.21	3	1.20		
*Frequency of craft beer consumption*					7.440	0.282
Less than once per month	68	31.78	79	31.60		
Once per month	32	14.95	36	14.40		
Less than once per week	38	17.76	61	24.40		
Once per week	31	14.49	38	15.20		
2–4 times per week	33	15.42	28	11.20		
Once per day	7	3.27	7	2.80		
More than once per day	5	2.34	1	0.40		

## Data Availability

The data presented in this study are available on request from the corresponding author. The data are not publicly available due to privacy restrictions.
